# Susceptibility of influenza A(H1N1)/pdm2009, seasonal A(H3N2) and B viruses to Oseltamivir in Guangdong, China between 2009 and 2014

**DOI:** 10.1038/s41598-017-08282-6

**Published:** 2017-08-16

**Authors:** Shan-shan Liu, Xiao-yang Jiao, Sheng Wang, Wen-zhe Su, Ling-zhi Jiang, Xin Zhang, Chang-wen Ke, Ping Xiong

**Affiliations:** 1 Department of Pharmaceutical Engineering, South China Agricultural University, Guangzhou, 510640 China; 2 Shantou University Medical College, Shantou, 515041 Guangdong China; 3 Key Laboratory of Molecular Biophysics of the Ministry of Education, College of Life Science and Technology, Huazhong University of Science and Technology, Wuhan, Hubei 430074 China; 4 Guangzhou Centre for Disease Control and Prevention, Guangzhou, 510440 China; 5 College of Life and Ocean Science, Shen zhen Key Laboratory of Microbial Genetic Engineering, Shenzhen University, Shen zhen, 518060 China; 6 Medical Key Laboratory for Repository and Application of Pathogenic Microbiology, Research Center for Pathogens Detection Technology of Emerging Infectious Diseases, Guangdong Provincial Center for Disease Control and Prevention, Guangzhou, 511430 P.R. China; 7 WHO Collaborating Centre for Surveillance, Research and Training of Emerging Infectious Diseases, Guangdong Provincial Center for Disease Control and Prevention, Guangzhou, 511430 P.R. China

## Abstract

Nasopharyngeal swabs were collected from patients through the influenza surveillance network of the CDC of Guangdong. All specimens between 2009 and 2014 were checked for influenza virus using MDCK cells and further subtyped. Of those collected, 542 H1N1pdm09, 230 A(H3N2)and 448 B viruses selected at random were subjected to fluorescence-based NAI assays. Viral RNA was extracted from resistant isolates, and their NA genes were amplified by RT-PCR. Alignment of nucleotides and amino acids was performed. We performed structural modelling and simulations of mutants using Modeller 9.x and AutoDock and analyzed conformations and binding affinities. All tested seasonal type B and H3N2 viruses from 2009 to 2014 remained sensitive to oseltamivir. However, there were five strains (out of 198 tested isolates acquired between June and September 2013) that were resistant to oseltamivir. Another three resistant strains were identified among isolates from March to April 2014. We found that 2013/2014 oseltamivir-resistant strains and 2012/2013/2014 oseltamivir-sensitive strains had all or some of the following mutations: N44S, N200S,V241I, I321V,N369K, N386 K and K432E. MutationsV241I, N369K, N386K and K432E, alone or in conjunction with H275Y, had a significant impact on the binding pattern and affinity of oseltamivir for neuraminidase, rendering neuraminidase less susceptible.

## Introduction

Influenza virus is a major pathogen that causes respiratory tract infections. Approximately 70–80% of influenza cases are caused by virus infection. Periodic influenza epidemics occur annually. Influenza virus infections continue to be a major cause of high morbidity and mortality worldwide, especially in children under 5 years of age and in the elderly^1^. Currently, loss of inhibitory activities by M_2_ ion channel inhibitors, such as amantadine and rimantadine, against human influenza H1N1, H3N2 and B lineages can be attributed to the antiviral resistance emerging worldwide. Thus, neuraminidase inhibitors (NAIs) have become the most recommended antiviral drugs for the treatment of seasonal influenza A and B infections for those most susceptible^2^.However, influenza viruses continually evolve under selective pressure, leading to antigenic drift and genetic reassortment among generations of viruses. This pressure causes influenza viruses to alter their susceptibility to antiviral drugs. Mutations within some key functional genes, including both single and double mutations, can significantly decrease susceptibility, leading to antiviral resistance. From 2007 to 2008, oseltamivir-resistant strains that possessed H275Y mutations in their NA proteins began to emerge within seasonal H1N1 viruses^3^. Before 2009, the percentage of oseltamivir-resistant viruses in all subtypes, including seasonal H1N1, was no more than 1%^4–6^. Surprisingly, the prevalence of oseltamivir-resistant seasonal influenza H1N1 viruses increased to almost 100% between 2009 and 2010, even in countries where people had never used oseltamivir^7^. In contrast, influenza A H3N2 and seasonal B viruses remained sensitive to NAIs^8^. The emergence and worldwide spread of oseltamivir-resistant seasonal H1N1 viruses attracted considerable concern^9, 10^. Furthermore, in 2009, seasonal H1N1 viruses resistant to both amantadine and rimantadine were detected in several countries^11, 12^, including in China, and particularly in HongKong and Guangdong^12–14^. In March and early April of 2009, pandemic A H1N1 2009 viruses emerged in Mexico and the United States, respectively, and then spread rapidly all over the world. Subsequently, oseltamivir-resistant seasonal A H1N1 viruses were replaced by pandemic H1N1 viruses. WHO received the first report regarding an oseltamivir-resistant H1N1pdm2009 isolate in July 2009^15^, and the H275Y mutation within NA was detected in the drug-resistant virus. During the 2009–2010 influenza season, although the use of oseltamivir increased more than ever, oseltamivir-resistant H1N1pdm2009 viruses were reported only sporadically^16, 17^, with no cases reported in China. China began using neuraminidase inhibitors later than many other countries, so there are few studies on antiviral resistance. Virological and epidemiological surveillance remains critical for the detection of evolving influenza viruses. In order to survey the susceptibility of circulating strains in Guangdong to oseltamivir and to determine whether amino acid sequence variations may have had an impact on antiviral susceptibility, the Guangdong CDC launched an unprecedented surveillance of influenza H1N1pdm2009, A(H3N2) and B viruses, and these circulating strains (from 2009 to 2014) were compared to the wild-types and reference viruses. The study provided valuable information regarding the prevention and management of Guangdong human influenza.

## Results

### Temporal distribution trend

**Figure 1:**
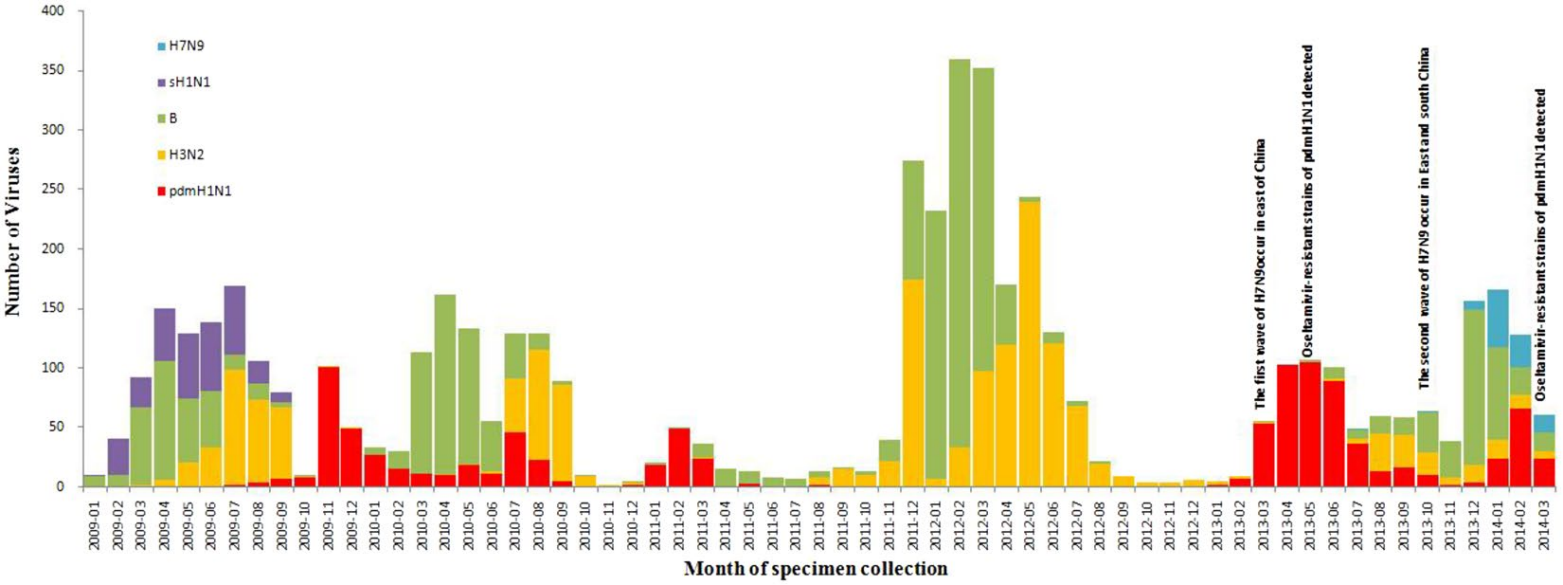
The number of influenza virus isolates by subtype in Guangdong, China from January 2009 to March 2014. The specimens of nasopharyngeal swabs were collected from patients through the influenza surveillance network of Centers for Disease Control and Prevention of Guangdong. All specimens during 2009 to 2014 were isolated for influenza viruses with MDCK and further subtype. Five color columns represented for the number of five subtype isolates which distributed in different month, respectively. The blue column stands for the number of H7N9 strains, the purple column stands for the number of seasonal A(H1N1) strains, the green column stands for the number of seasonal B strains, the yellow column stands for the number of seasonal A(H3N2) strains, the red column stands for the number of A(H1N1) pdm09 strains.

Amongst the 21 sentinel CDC centres in the Guangdong province, there were spring through summer seasonal influenza peaks every February through September. This seasonal outbreak is different from what occurs in Northern China and in the same latitude regions of America where the influenza activity peaks have occurred in winter months (December-January)^18, 19^. Furthermore, the predominant influenza virus types/subtypes based on isolates from clinical specimens were different for each season during the 63-month surveillance period. As shown in Fig. 1, seasonal H1N1 predominated during the 2009 influenza peak (accounting for 33.22% of isolates collected between February 1, 2009 and September 30, 2009). However, the A (H1N1) pdm2009 virus has since gradually replaced the seasonal H1N1 virus as of July 2009 (Fig. 1), after which there was a rapid rise in the number of cases, with the highest number of confirmed incidences in Guangdong. The virus also co-circulated with H3N2 and influenza B, but predominated during the 2009–2010 winter months (accounting for 95.19% of isolates collected November-January), the 2010–2011 winter months and the 2013 spring-summer months(accounting for 83.19% of isolates collected December 2010-March 2011 and 78.46% of isolates collected March-September 2013). Seasonal influenza B virus peaked during the 2009 and 2010 spring- summer months(accounting for 36.59% and 62.70% of isolates collected February-August of 2009 and 2010, respectively) and the 2011–2012 and 2013–2014 winter months (accounting for 73.35% and 61.15% of isolates collected November-March, respectively). During the study period, we observed that A(H1N1)pdm09 persisted as the predominant circulating strain between October 2009 and March 2011. New avian influenza A/H7N9 infections were first reported in east China in March but the first case was confirmed in Guangdong in July 2013. The outbreak of A/H7N9 infections occurred in Guangdong during the Spring of 2014. The numbers of isolated influenza viruses are listed in Table 1.

**Table 1:** Number of influenza viruses isolated in Guangdong, China from 2009 to 2014.

Year	Total isolates (n)	Number of influenza viruses by type(subtype)				
		Seasonal A (H3N2)	Seasonal A (H1N1)	A (H7N9)	pdm2009 A (H1N1)	B
2009	1083	293	299	0	173	318
2010	898	237	0	0	174	487
2011	513	231	0	0	100	182
2012	1610	730	0	0	1	879
2013	809	112	0	10	444	243
2014	355	35	0	90	114	116

### Antiviral susceptibility of influenza viruses

The susceptibility results for oseltamivir are summarized in Tables 2 and 3. In total, 230 seasonal A (H3N2) viruses, 542 A(H1N1) pdm2009 viruses and 448 seasonal B viruses selected at random were subjected to NAI assays. Based on the WHO’s definition of clinically oseltamivir-resistant, we found that no oseltamivir-resistant H1N1pdm viruses were isolated between 2009 and 2012. All seasonal influenza B viruses and H3N2 viruses tested from 2009 to 2014 were sensitive to oseltamivir. However, 5 of 198 tested H1N1pdm2009 isolates from 2013 were found to be resistant to oseltamivir. Similarly, 3 of 129 tested H1N1pdm isolates from 2014 were found to be resistant. Interestingly, some of the resistant variants had a more than 1000-fold increase in their IC_50_ values for oseltamivir compared to those of the sensitive viruses (Table 2).

**Table 2:** IC_50_ values for oseltamivir for the randomly tested isolates ($\overline{{\rm{x}}}$ ± SD, nM).

Year	Sensitive A(H1N1)pdm09		Resistant A(H1N1)pdm09		Seasonal A(H3N2)		Seasonal B	
	Number of virus strains	IC_50_	Number of virus strains	IC_50_	Number of virus strains	IC_50_	Number of virus strains	IC_50_
2009	117	—	0	—	0	—	0	—
2010	15	1.03 ± 0.25	0	—	98	0.24 ± 0.18	279	30.02 ± 13.40
2011	82	1.22 ± 1.00	0	—	27	0.37 ± 0.33	79	24.35 ± 12.42
2012	1	0.374	0	—	36	0.26 ± 0.13	10	23.74 ± 6.95
2013	194	0.49 ± 0.36	5	295.00 ± 126.54	21	0.33 ± 0.15	26	16.67 ± 11.45
2014	125	0.54 ± 0.44	3	355.67 ± 109.13	48	0.37 ± 0.28	54	17.68 ± 8.17

“—” = “Not determined”.

**Table 3:** The yearly distribution of viruses with highly-reduced sensitivity to oseltamivir from 2009 to 2014 in Guangdong.

Sample collection time	Number of resistant viruses/Number of tested viruses		
	Pandemic A(H1N1)	Seasonal A(H3N2)	Seasonal B
2009	0/117	—	—
2010	0/15	0/98	0/279
2011	0/82	0/27	0/79
2012	0/1	0/36	0/10
2013(June–Sept.)	5/198(2.52%)	0/21	0/26
2014(March–April)	3/129(2.32%)	0/48	0/54

“—”“ = “Not determined”.

The eight tested oseltamivir-resistant H1N1 pdm2009 viruses had IC_50_ values of 504 nM, 241 nM, 164 nM, 285 nM, 281 nM, 474 nM, 259 nM and 334 nM, respectively, as shown in Table 4. The oseltamivir-sensitive H1N1pdm viruses had IC_50_ values ranging from 0.374 nM to 1.22 nM. Seasonal A (H3N2) viruses had IC_50_ values ranging from 0.24 nM to 0.37 nM. Seasonal B viruses had IC_50_ values ranging from 16.67 nM to 30.02 nM.

**Table 4:** Susceptibility of eight influenza A(H1N1)pdm2009 viruses with the H275Y substitutions to oseltamivir(Guangdong, China isolates from 2013 to 2014)

Isolate name	Collection date	NA substitution	IC_50_(95%CI, nM)	IC50(95%CI, nM)
A/Guangdong- jiangmen/392/2013	2013-6-24	H275Y	H275Y	504 (10.1–25244)
A/Guangdong- jiangmen/393/2013	2013-6-25	H275Y	H275Y	241 (69–841)
A/Guangdong-zhongshan/402/2013	2013-7-09	H275Y	H275Y	164 (69.5–388)
A/Guangdong-zhongshan/414/2013	2013-7-09	H275Y	H275Y	285 (139–586)
A/Guangdong- zhanjiang/720/2013	2013-9-23	H275Y	H275Y	281 (145–433)
A/Guangdong-qingyuan/354/2014	2014-3-10	H275Y	H275Y	474 (15.4–14578)
A/Guangdong-meizhou/446/2014	2014-4-02	H275Y	H275Y	259 (145–463)
A/Guangdong-zhongshan/451/2014	2014-4-08	H275Y	H275Y	334 (133–836)

“CI” = “Confidience Intervals”.

### Analysis of the genetic characteristics of NA

To comprehensively analyse NA genetic variation, all the available NA sequences from NCBI databases were retrieved during the 2009–2014. The nucleotide IDs are as follows:FJ966084.1, GQ377078.1,GQ149688.1,GQ250162.1,CY103908.1,JF929787.1,CY1209333.1,CY120916.1,CY120928.1 and CY135173.1.The corresponding amino acid IDs are as follows:ACP4110 7.1, ACT36688.1, ACQ99635.1, ACR83539.1, AEX09177.1, AEF29967.1, AFN87942.1, AFN87925.1, AFN87937.1 and AGG20154.1. Analysis of NA polypeptide revealed that all circulating A(H1N1)pdm2009 osetamivir-resistant strains from Guangdong had a mutation at residue 275 within NA; that is, the histidine(H) was replaced by tyrosine(Y). When compared to the vaccine strain A/California/07/2009(KC7817851) and wild-type A/Mexico/4108/2009, the Guangdong A(H1N1)pdm2009 strains showed almost over than 98% homology, although NA protein in these strains contained multiple amino acid variations (Table 5). Specifically, between the Guangdong A(H1N1) pdm2009 isolates characterized during the 2009–2011 and A/California/07/2009 (KC7817851), the NA gene shared 99.57–99.79% amino acid identities. Between the Guangdong A(H1N1) pdm2009 isolates characterized in the 2012 and three referenced wild type strains, the NA gene shared 98.93% amino acid identities. Between the Guangdong A(H1N1) pdm2009 osetamivir-resistant strains in the 2013–2014 and three referenced wild type strains, the NA gene shared 98.29–98.72% amino acid identities. Apart from that, the NA gene mutation sites of Guangdong A(H1N1) pdm2009 resistant strains, circulating between the 2013–2014, were found to be consistent with that of the resistant strains from clusters cases reported in Sapporo of Japan, Louisiana and Washington of the United States, except few mutations like N44S and N386K. All of eight oseltamivir-resistant strains from Guangdong occurred N44S, N200S,V241I, H275Y and N369K mutations in common, among these, there are three strains possessing several other mutations like I321V,N386K and K432E. In addition, some amino acid substitutions at positions N44S, V106I,N200S,V241I,I321V,N369K,N386K and K432E were observed not only in 2013/2014 oseltamivir-resistant strains, but also in a few 2012/2013/2014 oseltamivir-sensitive strains.

**Table 5:** Characteristic amino acids of influenza A(H1N1)pdm2009 viruses isolated in Guangdong, China in 2009–2014(n = 18).

Isolated viruses	Amino acid position of the NA gene										
	19	34	44	106	200	241	275	321	369	386	432
*WT/A/California/04/2009	M	I	N	V	N	V	H	I	N	N	K
*WT/A/California/07/2009	M	I	N	V	N	V	H	I	N	N	K
*WT/A/Mexico/4108/2009	M	I	N	V	N	V	H	I	N	N	K
A/Guangdong/03/2009	M	I	N	I	N	V	H	I	N	N	K
A/Guangdong/5301/2009	M	I	N	I	N	V	H	I	N	N	K
A/Guangdong/202/2010	M	I	N	I	N	V	H	I	N	K	K
A/Guangdong/302/2010	M	I	N	I	N	V	H	I	N	N	K
A/Guangdong/102/2011	M	I	N	I	N	V	H	I	N	S	K
A/Guangdong/147/2011	M	I	N	I	N	V	H	I	N	S	K
A/Guangdong/1513/2012	M	I	S	I	S	I	H	I	K	N	K
A/Guangdong/310/2013	M	I	S	V	S	I	H	I	K	N	K
A/Guangdong/400/2013	M	I	S	V	S	I	H	I	K	N	K
A/Guangdong/392/2013	V	I	S	V	S	I	Y	I	K	N	K
A/Guangdong/393/2013	V	I	S	V	S	I	Y	I	K	N	K
A/Guangdong/402/2013	V	I	S	V	S	I	Y	I	K	N	K
A/Guangdong/414/2013	V	I	S	V	S	I	Y	I	K	N	K
A/Guangdong/720/2013	M	V	S	V	S	I	Y	V	K	K	E
A/Guangdong/354/2014	M	V	S	V	S	I	Y	V	K	K	E
A/Guangdong/446/2014	M	V	S	V	S	I	Y	V	K	K	E
A/Guangdong/451/2014	M	V	S	V	S	I	Y	V	K	K	E
A/Guangdong/452/2014	M	V	S	V	S	I	H	V	K	K	E
*MT/A/Sapporo/107/2013	M	V	N	V	S	I	Y	V	K	K	E
*MT/A/Sapporo/29/2014	M	V	N	V	S	I	Y	V	K	K	E
* MT/A/Louisiana/07/2013	M	V	N	V	S	I	Y	V	K	N	E
*MT/A/Washington/05/2014	M	V	N	V	S	I	Y	V	K	N	E
* MT/A/Newcastle/82/2011	M	I	N	I	N	I	Y	I	K	S	K
* MT/A/Newcastle/179/2011	M	I	N	I	N	I	Y	I	K	S	K

*Represents reference virus strains.

**Figure 2:**
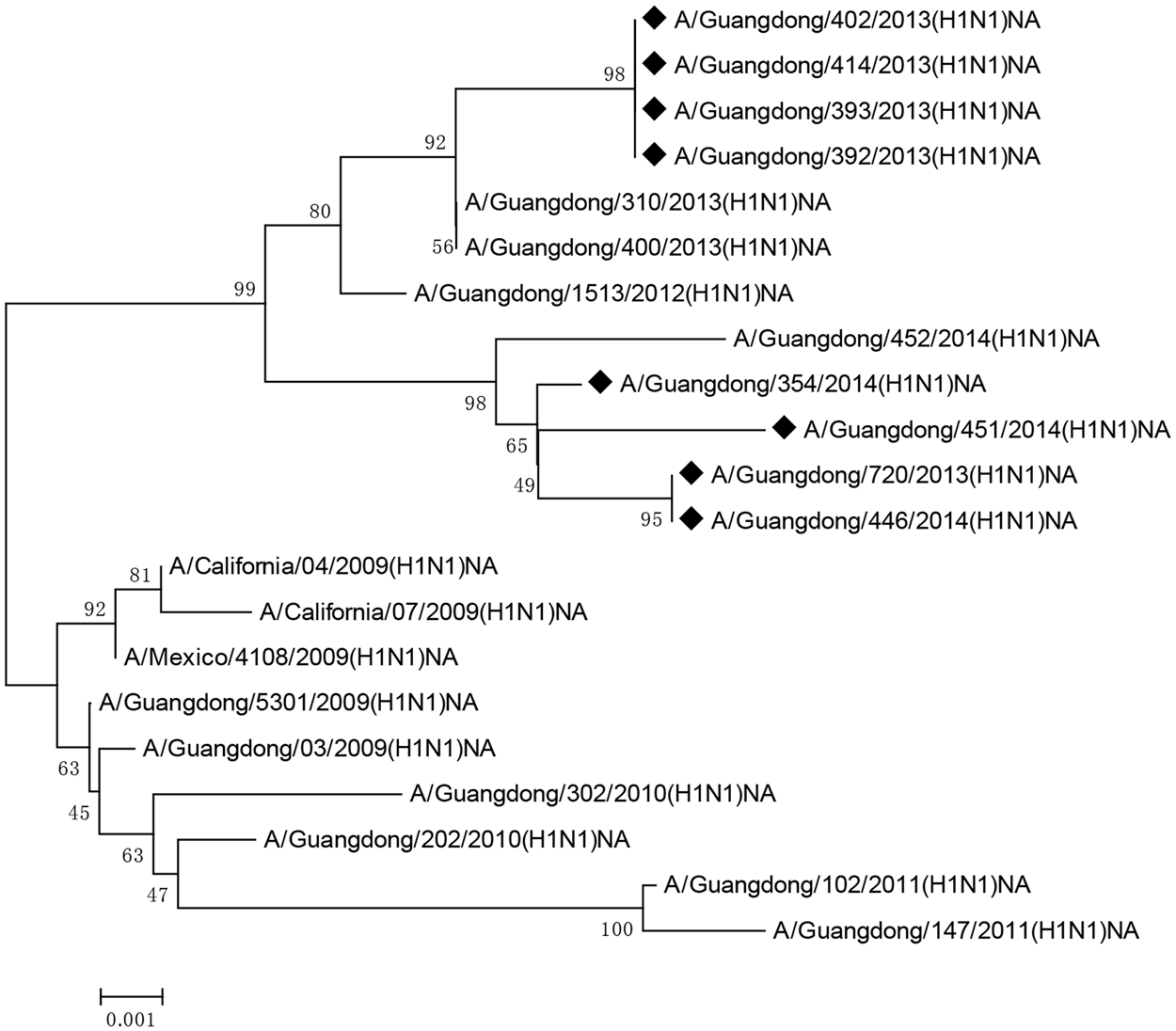
Phylogenetic analysis of NA sequences of influenza A(H1N1)pdm2009 oseltamivir-resistant strains from Guangdong, China.

In order to understand the origin and the genetic variation of Guangdong A(H1N1)pdm2009 strains circulating between the 2009–2014. Further, aligned sequences were used for phylogenetic analysis to group the most sequentially related NA protein with the referenced H1N1 (2009) wild-type strains from Mexico City and California clades. Phylogenetic tree was constructed using the MEGA 5.1 software with the neighbor-joining method. As has been shown in Fig. 2, the evolutionary analysis of NA gene demonstrated that four 2013 oseltamivir-resistant strains originated from the same H1N1 (2009) Human influenza strain, the rest of the 2013/2014 oseltamivir-resistant strains were derived from the same origin. To summarize, the mutant viruses of Guangdong cluster and the wild-type viruses of the Mexico/California may derive from a common ancestor. Therefore, the analysis suggested that the continuous and constant change in the NA region happened every year.

### Computational structural analysis of the NA mutations

To investigate whether amino acid mutations affect the susceptibility of A(H1N1)pdm09 viruses to oseltamivir or may be responsible for reinforcing the oseltamivir-resistant effect, a computational structural analysis of NA substitutions was conducted. In this work, homology modeling and mocular docking methods were applied. Firstly, highly homologous NA crystal structure was retrieved from PDB Database, and then NA mutations investigated were introduced separately or as cumulative combinations using FoldX, on this basis, accurate three-dimensional space structures of these mutants were constructed using Modeller 9.x software. After that, the mocular docking between ligand (oseltamivir) and recepters (NA mutants) were performed using AutoDock softwares. Finally, the molecular dynamics simulations and the interaction forces between oseltamivir and NA mutants were calculated using Amber 14 and GAMESS softwares.

No matter how, what we need to clarify was, in this study we found that many mutation sites existed in NA of sensitive and resistant strains from 2009–2014, but in fact just a few of them affect the susceptibility or resistance of NA to oseltamivir based on the systematic analysis of various NA structures. For example, the analysis found that Asn44 was far away from the binding pocket of oseltamivir, and all NA crystal structures reported had not the N-terminal peptide fragment. So in this study, the N-terminal peptide was not constructed into the stucture models, as well as N44S mutation. In addition, although the mutation of I321 residue happened in the NA variants, it had not remarkable effect to the whole conformation changes. A local hydrophobic core was composed of I321, I359, I374 and F387, which formed a very compact hydrophobic center. Even the I321V mutations existed in NA variants, the little difference of side chain between I and V can be adapted by hydrophobicity, the mutation did not force this hydrophobic core to change its conformation. So in this study, we pay more discussion on NA-V241I, N369K, N386K and K432E substitutions due to their potential role on susceptibility.

**Figure 3:**
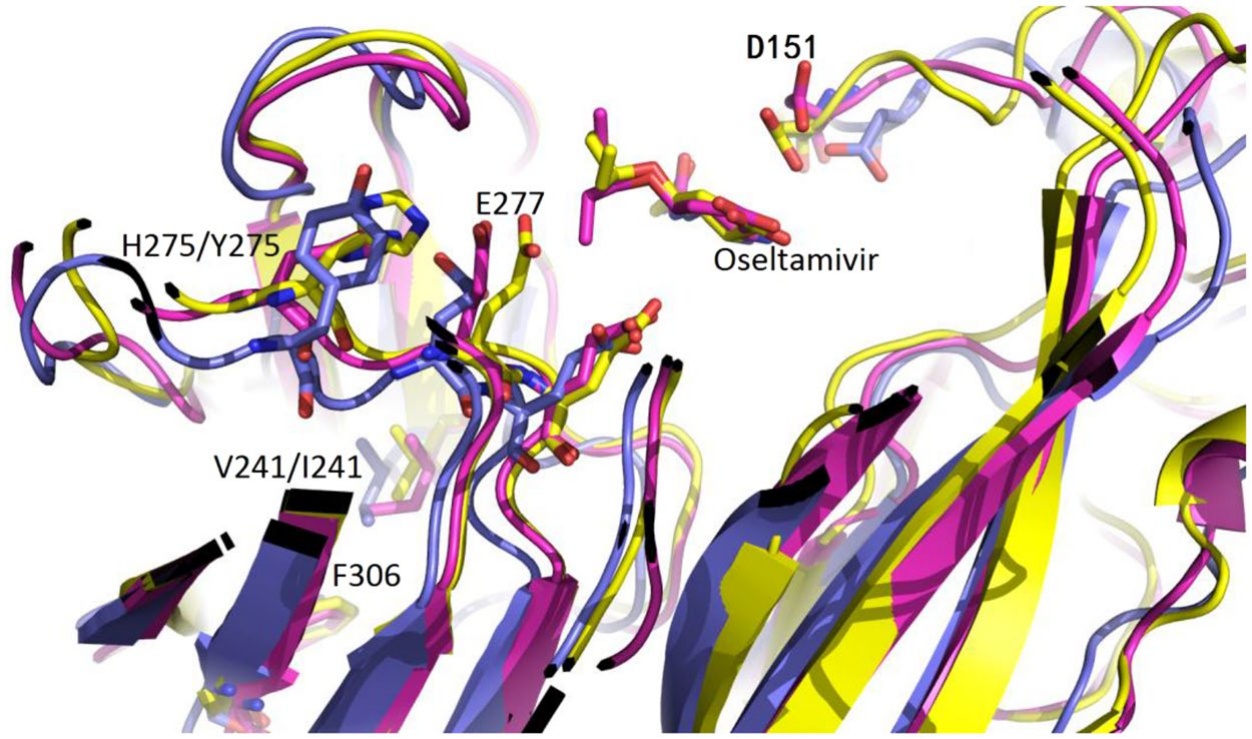
Alignment of V241I with H275Y. Using the wild-type NA(A/California/07/2009) as a reference structure, the two mutants were aligned together. Yellow: the reference NA. Magenta: NA with V241I alone. Slate: both V241I and H275Y.

For mutation V241I, sequence alignments analysis showed that V241I substitutions almost occurred in all resistant and sensitive strains circulating between the 2012–2014. Anyway, this mutation resulted in a longer side-chain and made it easier to interact with Phe306 through ahydrophobic effect, which shifted side-chains of His275 and Glu277 by 0.8 Å and 2.5 Å, respectively. At the same time, Asp151, located on the other side of the active pocket, shifted approximately 1.6 Å away from oseltamivir, and the distance between Asp151 and oseltamivir increased from 2.6 Å to 6.8 Å (Fig. 3). It was obvious that the latter shift strongly weakened the interaction between the -NH_2_ of oseltamivir and the COO^−^ of Asp151 (Fig. 3). If V241I existed in conjunction with H275Y, the side-chains of these residues would shift more than V241I alone. Thus, the binding pocket was elongated along the direction from H275 to D151, which makes the interaction of this key hydrogen bond weaker (Fig. 3).

**Figure 4:**
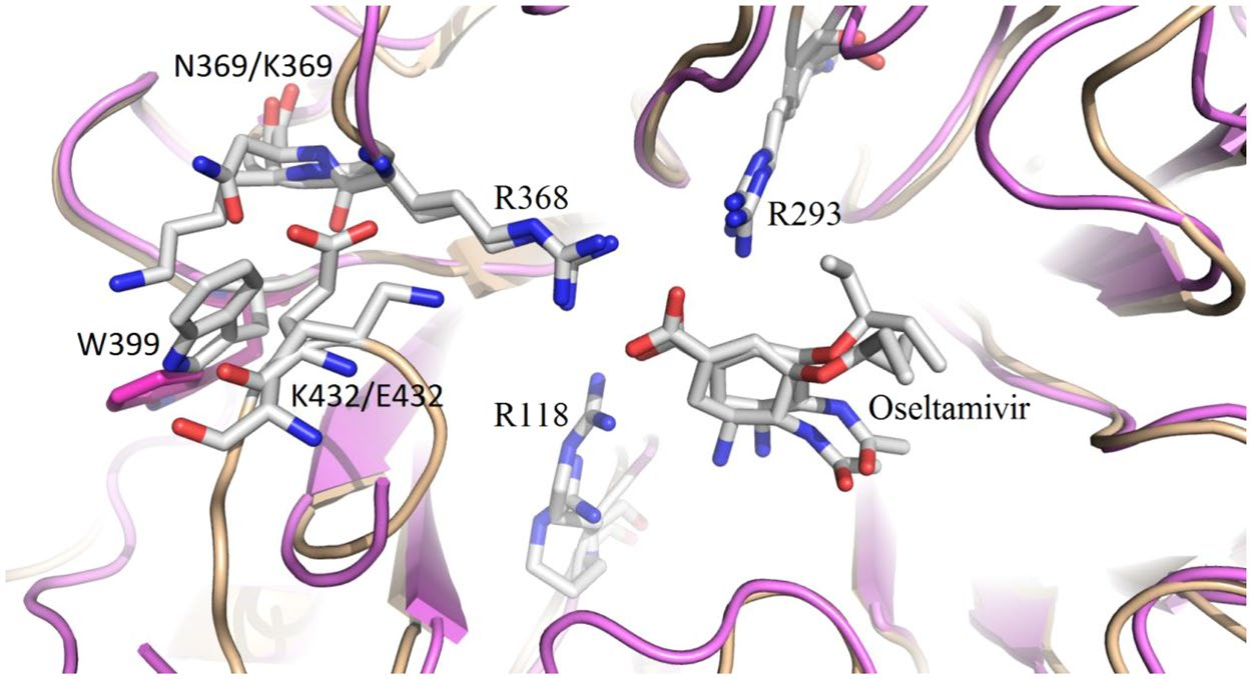
Alignment of the N369K and K432E mutations. The positively-charged domain of the binding pocket is split apart from the four positive residues(K432, R368, R293 and R118) into three as a result of the double mutations. The significant shifting of W399 is highlighted by the pink-coloured stick. Wild-type NA is denoted in violet, and the N369K/K432E NA is in white.

Another two important substitutions were N369K and K432E, as both single and double mutations. Along with Arg368 and Lys432, the N369K mutation made the local electrical density of NA more positive. The stronger positive electrostatic field made NA more susceptible to oseltamivir due to the negatively-charged COO^−^ side chain in this drug. If N369K and K432E both occurred in NA, this positive electrostatic field would be broken, and the mutated E432 would be able to form a salt bridge with K369 (Fig. 4). This pair of interactions was found in the simulated complex. The strong salt bridge effect even shifted the side chain of Trp399 outward approximately 4.4 Å, close to the K432E mutation. In addition, the double mutation (N369K and K432E) made the binding pocket contract slightly and the oseltamivir conformation rotate approximately 35.1 degrees (Fig. 4).

**Figure 5:**
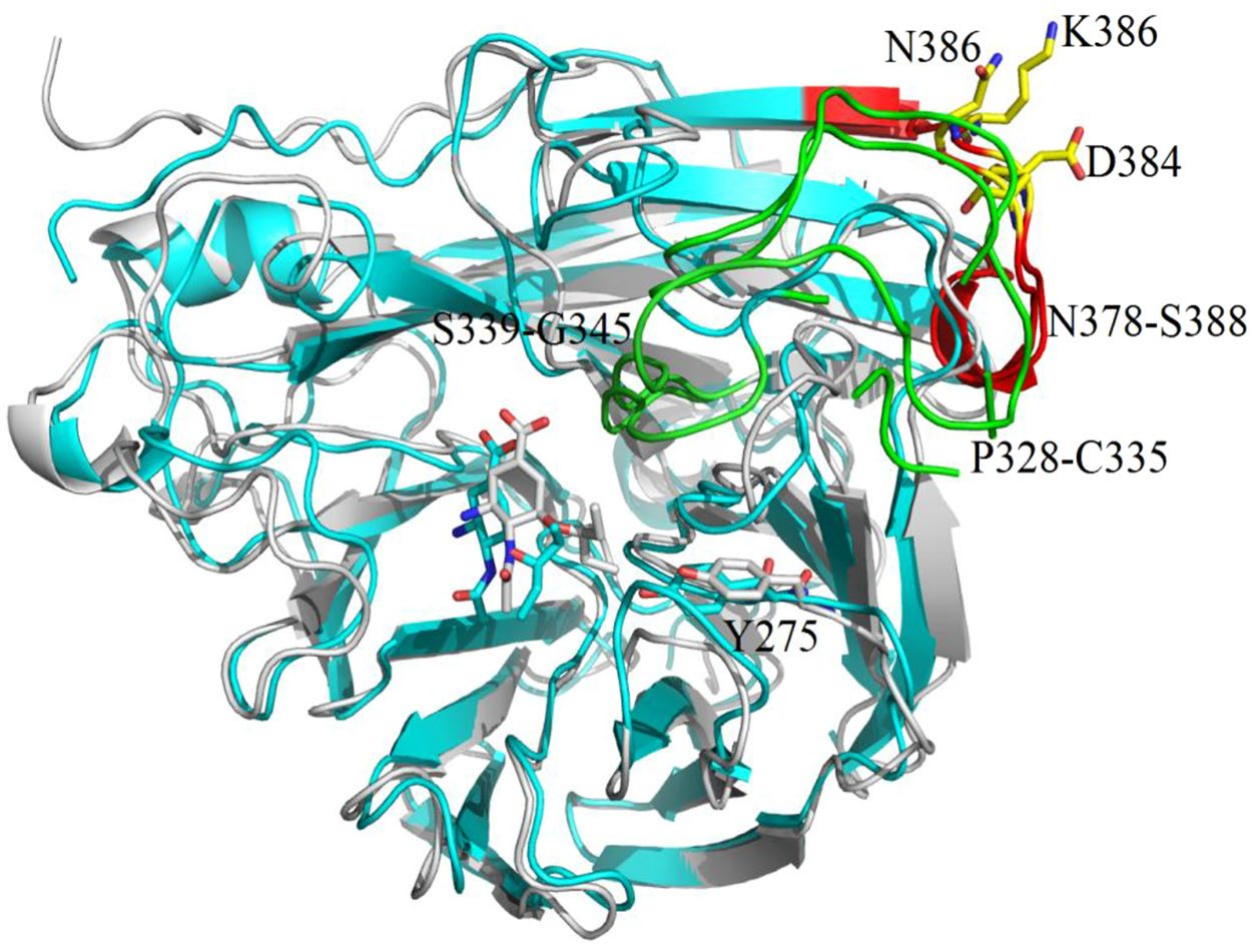
Loop rearrangement of NA resulting from the N386K mutation. The N386/K386 and D384 residues are labelled by colour element representations. The short loops are indicated by the red and green cartoons, which are directly affected by this mutation. Most loops located at binding sites conform to allow for a larger space, while the Y275 residue can shift a little ways from the bound ligand. These loop rearrangements will ultimately decrease the affinity of the ligand for NA.

For mutation N386K, significant changes within the side chain electrical properties would lead to a series of conformational changes. Although close to Asp384, the positive-charged K386 showed a stronger attraction to Asp384 and obvious conformational connections with the flexible loops of N378-S388 and P328-C335, as well as S339-G345. These connections could alter the susceptibility of oseltamivir to influenza viruses to a certain extent. As mentioned above, V241I made the binding pocket longer along residues H275 to D151. In addition, the V241I and N386K double mutation, which existed in most strains isolated from the Influenza Surveillance Network Platform of Guangdong Province, resulted in a synergistic effect within the binding pocket for oseltamivir. Thus, the N386K mutation, in combination with V241I, elongated the binding pocket while pushing several loops outward. The affected residues include R368, E119, D151 and R152, which formed stronger interactions with the -COO^−^, NH_2_ and -COCH_3_ groups of oseltamivir (Fig. 5).

## Discussion

In March and early April 2009, H1N1pdm2009 influenza broke out in Mexico and the United States, respectively, while pandemic A(H1N1)2009 viruses spread rapidly all over the world, including in China. In April 2009, the first H1N1pdm2009 influenza case from the Yuexiu district, Guangzhou city, Guangdong was diagnosed through a virological exam. As outbreaks continue, the world remains almost completely dependent on NAIs, oseltamivir first licensed in 2000, to prevent or treat influenza viruses infection﻿. However, resistance to antiviral drugs is known to emerge in humans after treatment and host-adaptive selection pressure. New resistant strains are generated through gene reassortment and transmission of mutated genes. Oseltamivir is a highly effective antiviral agent, and although there were few resistant viruses before 2008, during the 2007–2008 influenza season, an oseltamivir-resistant seasonal A(H1N1) influenza strain emerged in Europe and was further propagated, becoming the predominant circulating virus in just one year^7^. Interestingly, almost all seasonal H1N1 viruses around the world became resistant to oseltamivir in 2009. Oseltamivir was recommended within the Guangdong province of China for clinical treatment of severe influenza-like illnesses. No oseltamivir-resistant H1N1pdm2009 viruses emerged before June 2013, although H1N1pdm2009 viruses had become the predominant circulating viruses in Guangdong after replacing the seasonal influenza H1N1 since 2009. However, once again, a novel avian influenza H7N9 virus emerged in China in March 2013^20^. Given the high mortality of H7N9 infections, the early strategy to treat patients was oseltamivir. Although there was not one infection to be found in Guangdong before July 2013, using poultry and environmental surveillance in live poultry markets, we found that chickens had been infected with the H7N9 virus and were being sold in markets in cities within Guangdong in April and May 2013^21^. In May 2013, the public health bureau of Guangdong province asked the public health system to carry out enhanced ILI surveillance to discover the H7N9-infected individual early and prepare enough oseltamivir to treat potential cases. From the surveillance data, we found that oseltamivir-resistant A(H1N1)pdm2009 strains appeared only two months later. In other words, following the Guangdong province outbreak in 2009, oseltamivir-resistant A(H1N1)pdm2009 was not detected until June 2013. The surveillance results suggested that the emergence of resistant H1N1pdm2009 strains may be due to the increased use of oseltamivir after the H7N9 influenza outbreak. The correlation can be seen from Fig. 1. The fact of the increased use of oseltamivir after H7N9 epidemic also can be confirmed by the China official guidelines for diagnosis and treatment of H7N9 influenza.The guidance suggested treatment with antivirals for patients with confirmed, probable, or suspected avian influenza A(H7N9), and for individuals who are at increased risk for influenza-related complications. Moreover, the patients with influenza-like illness and normal or lower white blood cells, individuals who are at high risk for severe illness from avian influenza A(H7N9) should be given antiviral treatment as soon as possible. In a word, the guidelines emphasized early use of antiviral treatment for avian influenza A(H7N9), oseltamivir is strongly recommended for early treatment of suspected or confirmed avian influenza A(H7N9) or other severe influenza cases^22^.

Oseltamivir exerts the inhibitory effect through its active metabolite oseltamivir carboxylate(OC) recognizing and binding to the active domain of NA. Resistance to oseltamivir is caused primarily by the mutations of amino acid residues in NA domain which changed the binding affility. The H275Y mutation that confers resistance to oseltamivir has been detected in various human influenza N1 subtypes, including seasonal H1N1^9, 23–26^, highly pathogenic H5N1^27, 28^ and, more recently, A(H1N1)pdm2009 viruses^29–33^. It is well known that H275Y confers very strong resistance. Once the H275Y mutation appears, the sensitivity of the A(H1N1)pdm2009 clinical isolates to oseltamivir can decrease by 1466-fold when compared to that of wild-type^25^. There have been many studies on the resistance mechanism of NA-H275Y mutation A(H1N1)pdm2009 strains to oseltamivir^34, 35^.

In this study, we surveyed the susceptibility of circulating strains in Guangdong during the 2009–2014 to oseltamivir by phenotypic and genotypic test. As a result, we detected out eight osetamivir-resistant A (H1N1)/pdm2009 viruses from the 2013/2014 isolates. It was also the first time that osetamivir-resistant strains were discovered in Guangdong, China in June 2013 since A(H1N1)/pdm2009 worldwide pandemic. Further findings presented that all of eight oseltamivir-resistant strains occurred N44S, N200S, V241I, H275Y and N369K mutations in common, among these, there are three strains possessing NA–I321V, N386K and K432E mutations. In addition, when compared to two wild type strains, like as A/California/04/2009 and A/Mexico/4108/2009, a few 2012/2013/2014 sensitive strains have happened N44S, N200S, V241I, N369K and N386K mutations.

Anyway, it have been previously reported that A(H1N1)pdm09 mutation viruses possessed N44S, V106I, N200S, I223R/V, V241I, S247N, N369K and N386K substitutions in the NA gene. With regard to these site mutations, there also have been some study reports on their potential effects^36–39^. For example, NA–S247N and I223R/V mutations in A(H1N1)pdm09 influenza viruses can confer reduced susceptibility to oseltamivir. A novel A(H1N1)pdm09 variant containing NA–S247N mutation was found mainly in the Asia-Pacific area in recent years. This mutation reduces sensitivity and confers extremely high resistance to oseltamivir in combination with the H275Y mutation when compared to resistance conferred by the H275Y mutation alone^40, 41^. Likewise, when compared to the wild-type strains, clinical isolates with NA–I223R mutations had an approximately two-fold reduced affinity for the substrate; the isolates containing H275Y- and I223R- also showed decreased susceptibility to oseltamivir(246-fold)^42, 43^. In addition, the other NA substitutions, like as V241I, N369K and N386K, which were presented in the oseltamivir-resistant strains studied here, may offset the destabilizing effect of the H275Y substitution. Particularly, the N369K mutation, which was computationally predicted to cause the largest change in NA protein stability, has previously been shown experimentally to increase NA surface expression and activity in combination with H275Y^44^. In this work, we applied computational analysis methods to discuss whether the binding affinity between NA and oseltamivir will happen to change when NA occurred V241I, N369K, N386K and K432E mutations, alone or in combination with H275Y, and further have a impact on susceptibility of A(H1N1)pdm09 strains to oseltamivir. Anyway, it stands from another new perspective to analysis and interpret the potential correlation between phenotype and genotype.

However, the computational structural analysis showed that some amino acid residues, like as I34V, N44S, I106V, N200S and I321V, are not located in the oseltamivir-binding domain of NA. So in this work, the analysis primarily focused on mutations within the binding domains, including V241I, N369K, N386K and K432E. The results showed that the binding pocket of NA became narrower upon mutation of N369 and K432 (N369K and K432E), so that the binding angles changed approximately 35.1 degrees, which led to decreased binding of oseltamivir to NA. The V241I mutation almost was presented in all resistant and sensitive strains circulating between the 2012–2014. If NA–V241I mutation emerged in conjunction with H275Y, they will generate the synergy effect in the highly reduced sensitivity of viruses to oseltamivir. In addition, the binding ability of oseltamivir to NA also decreased when mutations V241I and N386K were present. On balance, the obtained results revealed that V241I, N369K, N386K and K432E mutations may alter the susceptibility of A(H1N1)pdm09 variants to oseltamivir.

Apart that, the current study revealed that the NA–V241I/N369K/N386K/K432E mutation in conjunction with the H275Y substitution had emerged in the 2013/2014 resistant variants in Guangdong, China. Furthermore, sensitive strain, like as A/Guangdong/452/2014 strain, also possessed NA–V241I/N369K/N386K/K432E quadruple mutation. Noticeably, A/Guangdong/452/2014 strain have a relative higher IC50 (1.44 nM) than the IC50 average value 0.54 nM of sensitive strains. We thought that this should not be occasional. Although A(H1N1)pdm09 viruses with the mutations NA–V241I, N369K, N386K and K432E are not clinically defined as oseltamivir-resistant strains according to the definition of the WHO, its greater reduction in susceptibility to oseltamivir (2.67-fold increase in IC_50_) suggested that these substitutions may have contributed for the significantly higher IC50 values obtained, compared to wild-type viruses.

In conclusion, the current studies suggest that V241I, N369K, N386K and K432E mutations, alone or in combination with H275Y, may change the binding affinity between oseltamivir and NA, and further have a impact on susceptibility of A(H1N1)pdm09 strains to oseltamivir. So the emergence of such variants should be carefully monitored for public health concerns. It’s very obvious that our study results in general were coincided with the current reported views. Anyway, the obtained results will contribute to supplement scientific literature on the susceptibility of predominant circulating influenza virus to oseltamivir, particularly, for the global assessment of A(H1N1)pdm09 virus susceptibility profile and baseline level to NAIs.

## Materials and Methods

### Clinical specimen collection and influenza virus subtyping

In our experiments, influenza A(H1N1)pdm2009 and seasonal A(H3N2) and B viruses were obtained through the influenza-like illness(ILI) surveillance system of the Guangdong Provincial Centre for Disease Control and Prevention. This network is composed of 21 representative municipal CDCs. Each CDC selected one or several sentinel hospitals located in their respective cities. These hospitals were selected based on regional representation and demonstrated capacity to conduct ILI surveillance. Sentinel hospitals collected nasopharyngeal swabs from patients according to a standard procedure, and the swabs were kept in viral transport medium and stored at 4 °C for up to 24 hours prior to analysis. Virus isolation was prepared in physical containment level 2 biosafety facilities by culturing in MDCK cells and embryonated SPF (specific pathogen-free) eggs. Influenza virus subtyping was performed according to the serological methods of the National Standard Operating Procedures (SOP).

### Drugs

Oseltamivir (Ro64802002) were kindly donated by F. Hoffmann-La Roche Ltd., Basel, Switzerland. The drugs were dissolved in MilliQ water to a final concentration of 5 μM and stored at −20 °C.

### Influenza virus isolation with MDCK cell lines

In this experiment, MDCK (ATCC, USA) cell lines passaged 3–50 times were used. MDCK cells(10^5^/ml) were seeded in T25 flasks with DMEM(Hyclone, USA) containing 10% Fetal Bovine Serum(FBS, Hyclone, USA) and 1% penicillin-streptomycin(Sigma, USA) in a final volume of 6 mL and then incubated at 37 °C in a humidified air atmosphere with 5% CO_2_ for 48 hours. When the cells reached 70–90% confluence, the culture medium was discarded, and the cells were washed three times with Hank’s solution. After the culture medium was removed, an appropriate amount of clinical specimen was added into the flask with a sterile pipette. Subsequently, the flasks were gently shaken and then incubated at 37 °C in a humidified air atmosphere with 5% CO_2_ for one or two hours to allow for virus absorption. The infected cells were again washed twice with Hank’s solution after removing the inoculum. Finally, the prepared virus growth medium was added to the flasks, and the cells were incubated at 33–35 °C. The cell culture was observed daily for cytopathic and morphological changes using an inverted light microscope. The culture supernatant was harvested when 75–100% of the infected cells appeared to be cytopathic and stored at −80 °C.

### NAI susceptibility

A fluorescence-based neuraminidase inhibition assay using the fluorescence substrate 2′-(4-methylumbelliferyl)-α-D-N-acetyl neuraminic acid (4-MU-NANA; Sigma, St. Louis, MO) was performed in accordance with international Standard Operating Procedures(SOP) to measure influenza virus neuraminidase (NA) activity in the presence of oseltamivir.Hydrolysis of the α-ketosidic bond in 4-MU-NANA by NA releases the fluorescent indicator 4-MU. Oseltamivir was added at the intended concentrations. The NAI susceptibility of influenza viruses was characterized by using oseltamivir concentrations that inhibited NA activity by 50%(IC_50_) as described previously^45^. As defined by the WHO, influenza A viruses with normal inhibition (NI) have a < 10-fold increase in IC_50_.Viruses with reduced inhibition (RI) have a 10- to 100-fold increase in IC_50_. Viruses showing highly reduced inhibition (HRI) are those with a > 100-fold increase in IC_50_, these viruses are considered clinically resistant. Oseltamivir-resistant viruses were defined as influenza viruses having IC_50_ values greater than 100 nM^9^.

### Primer design

NA, HA and M2 primers for H1N1pdm2009 were designed according to the results of the NAI susceptibility assay. The genes for NA, HA and M2 were amplified with 2–3 overlapping PCR segments to ensure that good quality sequencing data could be obtained from both directions on one segment. The specific primers were as follows: NA: 5′-ATGAATCCAAACCAAAAG-3′(forward), 5′-TTACTTGTCAATGGTAAATGG-3′(reverse); HA: 5′-ATGAAGGCAACA CTAGTAG-3′(forward), 5′-TTAAATACATATTCTACACTG-3′(reverse); M2:5′-AGCACT ACAGCTAAGGCTATGG-3′(forward), 5′-AGTAGAAACAAGGTAGTTTTTTACTC-3′ (reverse). The product sizes of NA, HA and M2 were approximately 1410 bp, 1710 bp and 572–1027 bp, respectively. All primers were synthesized by Sangon Biotech(Shanghai, China).

### Viral RNA extraction and gene amplification

Viral RNA was extracted from A(H1N1)pdm2009 virus cultures using QIAamp® Viral RNA Mini Kit (QIAGEN, Hilden, Germany) according to the manufacturer’s instructions. RNA was dissolved in 20 μL RNase-free ddH_2_O (QIAGEN, Germany) and quantified using a NanoDrop ND-100 device (Thermo Fisher Scientific, USA). Reverse transcription PCR(RT-PCR) of the sample genes was conducted with a one-step RT-PCR Kit (QIAGEN, Hilden, Germany) using a ThermoScript™ RT-PCR System and primers as designed previously. The genes were amplified in a 25 μL volume using an optical 96-well tray. The reaction mixture consisted of 5 μL of mRNA, 12.5 μL of 2 × Mix buffer, 1 µL of forward primer, 1 µL of reverse primer, 0.5 μL of Taq high fidelity DNA polymerase, and 5.0 μL of RNase-free ddH_2_O in PCR tubes. PCR cycling conditions were as follows: one cycle of 30 min at 50 °C and 2 min at 94 °C; 40 cycles each of 30 seconds at 94 °C, 30 seconds at 53–57 °C and 50–90 seconds at 72 °C; and a final extension at 72 °C for 10 seconds. The amplicons were confirmed by QIAcel advanced capillary electrophoresis(QIAGEN, Germany) and purified by SAP(USB ExoSAP-IT PCR Product Clean-Up, 5000 Reactions, Affymetrix, Canada).

### Sequencing and sequence analysis

Prior to sequencing, Sanger PCR(chain termination method) was performed using an ABI PRISM BigDye Terminator V1.1 Cycle Sequencing Kit (Applied Bio-systems, Foster City, CA, USA) and primers designed previously, with the amplicon as a template. The reaction mixture consisted of 0.5 μL of BigDye, 2 μL of 5× sequencing buffer, 1 µL of primer, 1 μL of template, and 5.5 μL of RNAase-free ddH_2_O in PCR tubes. PCR cycling conditions were as follows: one cycle of 1 min at 96 °C; 25 cycles of 10 seconds at 96 °C and 15 seconds at 50 °C; and a final extension at 60 °C for 4 min. The reaction products were purified using a BigDye X Terminator Purification Kit(Applied Biosystems, USA) and run on an ABI 3100XL sequencer (ABI, USA). Sequencing results were analysed using an ABI PRISM 3100 Genetic Analyzer (Applied Bio-systems, Foster City, CA, USA). The nucleotide and amino acid sequence data for NA of influenza A(H1N1)pdm2009 were retrieved from NCBI GeneBank and NCBI Influenza Sequence Database (http://www.ncbi.nlm.nih.gov/genomes/FLU/FLU.html), using NA reference sequences A/California/07/2009/H1N1, A/California/04/2009/ H1N1 and A/Mexico/4108/2009/H1N1. Alignment of nucleotide and amino acid sequencing data and phylogenetic tree were performed using MEGA software 5.1 (Centre for Evolutionary Functional Genomics, Tempe, AZ) and BioEdit 7.0.9.0. In addition, sequence analysis of sensitive viruses was performed using SNP pyrophosphate detection.

### Modelling and analysis of NA mutations

To understand a correlation between phenotype and genotype, and reveal whether amino acid substitutions at residue V241, N369, N386 and K432 of the neuraminidase (NA) protein in A(H1N1)pdm09 variants may alter susceptibilities to oseltamivir. So by the computational structural analysis methods, we analyzed A(H1N1)pdm09 variants from Guangdong, China during the 2009–2014. In the work, We performed homology modeling and mocular docking using Modeller 9.x and AutoDock software. Based on the results of NA sequencing and NAI susceptibility assays, the changes of binding affinities and conformations between NA and oseltamivir were analyzed, by taking that of wild-type A/California/04/2009 pandemic strains as a reference. Here, 4 structures with the PDB IDs 4B7Q, 3NSS, 4QNP and 4B7R were used as templates to construct the mutated NA proteins (V241I, H275Y, I321V, N369K, N386S, N386K and K432E, either alone or in combination). All modelled complexes were placed into similar physiological environments, and standard molecular dynamics (MD) simulations were performed to obtain stable conformations. The Amber 14 suite was used for all MD simulations, with the force-field parameters of oseltamivir calculated using Gaussian 09.

### Statistical Analysis

SPSS 13.0 (SPSS Inc. Chicago, IL, USA) was employed for statistical analysis. IC_50_ values were calculated using SPSS 13.0 software. Quantitative data are presented as the mean ± standard deviation(SD). The comparison of IC_50_ values was conducted using analysis of variance(ANOVA) with a *t-*test or *Levene* test. Levels of significance were set at P < 0.05.
